# Perceptions of the Body and Body Dissatisfaction in Primary Education Children According to Gender and Age. A Cross-Sectional Study

**DOI:** 10.3390/ijerph182312460

**Published:** 2021-11-26

**Authors:** Rubén Navarro-Patón, Marcos Mecías-Calvo, Silvia Pueyo Villa, Vanessa Anaya, Mariacarla Martí-González, Joaquín Lago-Ballesteros

**Affiliations:** 1 Facultad de Formación del Profesorado, Universidade de Santiago de Compostela, 27001 Lugo, Spain; ruben.navarro.paton@usc.es (R.N.-P.); joaquin.lago@usc.es (J.L.-B.); 2 Facultad de Ciencias de la Salud, Universidad Europea del Atlántico, 39011 Santander, Spain; mariacarla.marti@uneatlantico.es; 3 Facultad de Ciencias Sociales y Humanidades, Universidad Europea del Atlántico, 39011 Santander, Spain; silvia.pueyo@uneatlantico.es (S.P.V.); vanessa.anaya@uneatlantico.es (V.A.)

**Keywords:** body perceptions, body dissatisfaction, school children, physical education, primary education

## Abstract

Body image (BI) is a trending topic of study since health problems derived from a negative perception of the body are increasing and affecting people of all ages, with an increasing incidence among children from the age of eight. The objective of this study was to evaluate the current perception of the body against the desired body and the degree of body satisfaction of Galician primary education students. A total of 355 students (167 boys (47%)) between 9 and 12 years old participated (mean = 10.53; SD = 0.84). Sociodemographic data (sex, age, height, and weight) were collected, and the Figure Rating Scale was used. There are statistically significant differences between boys and girls in the current perceived figure (*p* = 0.003) and in the desired figure (*p* < 0.001). Depending on age, the differences were in current (*p* = 0.010) and desired (*p* = 0.021) body perception. In conclusion, boys perceive themselves as having a larger figure than girls do, but this perception is far from reality according to the body mass index. For the desired figure, both boys and girls want to be slimmer, but girls want a slimmer figure. Regarding age, the current perceived figure size increases with age as it increases in those students dissatisfied with their body.

## 1. Introduction

Perceptions about the body and physical care have increased the relevance and current tendency of society to pay attention to and have interest in the body [1]. This can be seen in most of our society and culture, since both have collaborated in the dissemination of a perception of the body based on the slim and slender figure of women [2,3,4] and male musculature development [1,3,5,6,7] and the struggle in achieving it, leading to alterations of the perception of the body, such as body dysmorphic disorder, in both men and women [8].

Bodies have been praised for many centuries. However, in today’s society, an excessive and obsessive value is placed on body aesthetics, physical care, and body image [9,10]. Therefore, this new social situation becomes a problem [1], the consequence of which is a confrontation between the real appreciation of the body and that of the legitimate or desired body [11,12].

On many occasions, the terms “body image”, “negative body image”, “body dissatisfaction”, “positive body image”, and “body satisfaction” are often confused and used interchangeably. 

Body image is a nonspecific, broad term that refers to a series of cognitive, affective, perceptual, and behavioral elements that describe the various ways in which individuals relate to their physical self. Positive and negative appearance generally refers to healthy/adaptive versus unhealthy/maladaptive relationships with the physical self. In this sense, body image is defined as a subjective phenomenon that must be considered within the cultural context in which people with body dissatisfaction find themselves [9]. This mental image can have positive or negative connotations, which will affect our emotional, physical, and social well-being in some way [13]. This may be conditioned by intrinsic factors, such as physical changes [6] or body composition [14], or by extrinsic factors, such as the media [15], due to socially established norms of beauty [1,2,13,16]. Even public health services that relate negative stereotypes of body size and people with fat and health status can affect body image [17,18].

Body satisfaction or dissatisfaction represents components of positive or negative perceptions of the body and often refers to the positive or negative evaluation of a person’s general appearance [19]. Thus, body dissatisfaction is a psychological symptom caused by a self-image that deviates from an ideal perception of the body [7]. In this sense, numerous authors, such as Bornioli, et al. [20], Kahan, et al. [21], and Schuck, et al. [22], have associated body dissatisfaction with health consequences, such as decreased physical activity, or related to psychosocial aspects, such as weight changes, depression, anxiety, low self-esteem, and eating disorders [6,7,10,23,24,25], which is currently considered as a public health problem [26]. 

Body image is a multidimensional theoretical construction that begins in childhood [6] and continues into preadolescence and adolescence [10,27], beginning between 9 and 12 years of age in girls and between 10 and 13 years in boys [6]. Body dissatisfaction can begin at ages prior to puberty [6,22,28,29]. Mancilla et al. [28] confirm that preschool children are competent to assess their physical proportions and contrast different weights. In addition, they are aware of the western preference for slenderness and begin to express their concerns about it. When children grow and interact with others, they begin to compare themselves, especially in appearance (for example, little children desire to be bigger). Due to this, from the age of 6, the shape of the body becomes more important, since 40%–50% of children from 6 to 12 years old admit dissatisfaction with the size or shape of some part of their body [30,31]. Before puberty, there are extremely important body, brain, sexual, emotional, and social changes [6], and consequently, this is when more dissatisfaction with one’s own body can occur [32,33]. Therefore, this is a critical stage in body image construction [22,29], so interventions aimed at body dissatisfaction should be carried out from early adolescence [34].

Problems in the perception of the body during childhood will affect the future physical and mental health of children in the short and long term [15]. For this reason, and to understand the development and formation of perceptions of bodily attitudes, primary school is an important stage for the study of these phenomena [12,35,36], since it is considered a conductive environment for the implementation of health prevention programs integrated into existing curricula, allowing for wide dissemination [37,38].

Although historically body image research has focused on girls and women, certain aspects of the ideal body have changed over time [39], so the scientific literature shows that discontent, weight, and concern for the body are present in men and women [28,40], although they differ according to gender [41]. It is not a phenomenon linked to only female gender [42,43]. Despite this, girls show a higher proportion of dissatisfaction than boys do [3,7], perhaps because girls are more sensitive to negative stimuli than boys are [7,44] and therefore, can be considered a vulnerable group with respect to manifestations related to eating disorders [22,45]. They are also more influenced by the media, which generates a body appearance dissatisfaction [3,7]. On the other hand, male issues are not as well researched in relation to body dissatisfaction [46]. Therefore, body dissatisfaction could be approaching an equal prevalence between boys and girls [29].

For all the above, and for the knowledge of the authors, this is the first study in the Northwest of Spain aiming to evaluate the current perceptions of the body and desired body by Primary Education students through the Figure Rating Scale, and body dissatisfaction in contrast to the body mass index (BMI), and to examine the factors that may influence it (age and gender). These objectives are addressed through the following research questions: (1) Are there differences in current perceptions of the body and desired body based on gender in schoolchildren aged 9 to 12 years old? (2) Are there differences in current perceptions of the body and desired body depending on the age of schoolchildren from 9 to 12 years old? (3) Is there body dissatisfaction in schoolchildren aged 9 to 12 years old? Is it higher in girls? Is it higher in boys?

## 2. Materials and Methods

### 2.1. Study Design

A non-experimental, descriptive-observational, comparative cross-sectional design was carried out [47].

### 2.2. Participants

Selecting the sample for the research was nonprobabilistic and for convenience, with subjects selected due to geographical proximity and voluntary participation. A total of 4 educational centers from cities in Galicia (Spain) (A Coruña (1), Lugo (1), Ourense (1), and Pontevedra (1)) were invited. According to the total number of students enrolled in Galician educational centers [48], with a margin of error of 5% and a confidence level of 90%, it would be necessary to reach a sample size of 271 individuals; for this reason, 400 schoolchildren were accessed. Finally, a total of 355 Primary Education students participated. Twenty-five were excluded for not presenting the informed consent from their parents or legal guardians, and 20 for presenting incomplete data in the measurements.

Apparently healthy adolescents with no known psychotic illnesses or eating disorders were included in the study. Participants with known mental health problems and physical disabilities were excluded.

### 2.3. Measurements

Age, gender, and grade were collected through self-reports at the beginning of the Figure Rating Scale [49]. This scale has great reliability and validity [49,50,51] and is adapted to the Spanish context [1,52,53]. The height and weight of the participants were measured in light clothing and without shoes in an isolated room, to preserve the privacy of the schoolchildren, by two of the researchers, once the scale was completed. The BMI (Kg/m^2^) was calculated based on the previous variables. Subsequently, the variable was recoded following the WHO indications (0 = underweight person; 1 = normal weight person; 2 = overweight person; 3 = obese person) [54].

**Figure 1:**
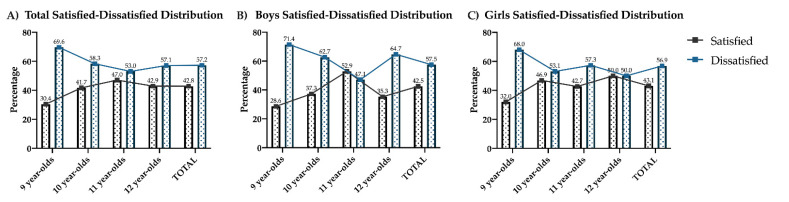
Body image satisfaction and dissatisfaction of boys and girls according to age. (**A**) Total distribution (boys and girls); (**B**) boys Distribution; (**C**) girls Distribution.

**Figure 2:**
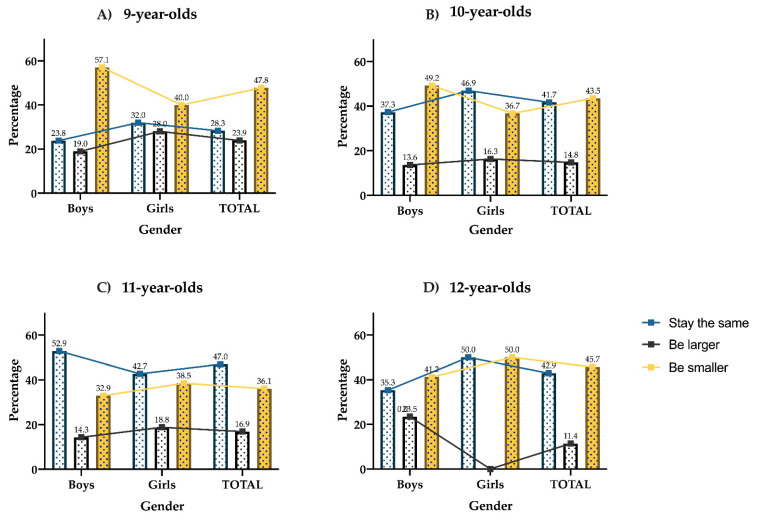
Desire to stay the same, be larger, or smaller of all schoolchildren who participated in this research and according to age. (**A**) 9-year-olds; (**B**) 10-year-olds; (**C**) 11-year-olds; (**D**) 12-year-olds, and gender (boys and girls).

For the BI analysis, the Figure Rating Scale [49] was used, which consists of 9 silhouette figures that gradually increase in size, from very thin (a value of 1) to very large people (a value of 9). Figures 1 and 2 correspond to people with low weight, Figures 3 and 4 to normal weight, Figures 5 to 7 to large people, and Figures 8 and 9 to very large people. This scale was completed two times by the students (the first to indicate how they look today by answering the question of “choose the figure that reflects how you think you look yourself” (current perception of the body), and the second to indicate how they would like to be answering the question “choose your ideal figure” (desired body)).

The results offer 2 measures: the current perception of the body, the desired body, and from them, the discrepancy (desired–perception) was calculated, which is interpreted as a measure of body (dis)satisfaction [55]. If the discrepancy is equal to 0, the subject is satisfied with their body; if the discrepancy has a positive value, the subject wants to be larger, and if the discrepancy has a negative value, the subject wants to be smaller. In addition, the following was used to calculate the degree of (dis)satisfaction:–desired body-current perception of the body. The following ranges were also established (that is, satisfied (difference = 0), slightly dissatisfied (difference ± 0.01–1); moderately satisfied (difference ± 1.01–2), and very dissatisfied (difference ± 2.01)] [11].

### 2.4. Procedures

To conduct this study, researchers contacted 4 potential participating centers and provided detailed information about the study and its purpose. Once their willingness to collaborate in the study was confirmed, permission was requested from the parents and/or legal guardians of the participating schoolchildren. Consequently, only those students with the corresponding written authorization from their parents or legal guardians participated in the study. Likewise, the research was approved by the ethics committee of the Educa platform (protocol code 22019, approved 30 January 2019) in accordance with the Declaration of Helsinki.

The data collection and the application of the scale were performed by the researchers in the facilities of the participating centers through a Physical Education session. The scale was administered in the Physical Education class without the presence of the subject teacher to avoid interference in the performance of the same for an average time of 10 min. The researchers proceeded to offer a brief initial explanation on the content of body shapes and answer any existing questions.

The height and weight measurements of the students were carried out once they had completed the Figure Rating Scale with comfortable clothing and barefoot in an isolated room to preserve the privacy of the schoolchildren.

### 2.5. Statistical Analysis

Descriptive values (mean and standard deviation), frequencies, and percentages were calculated for the characterization of the participants in this research. To check the homogeneity of variance, the Levene test was used. 

The current perception of the body, the desired body, and the discrepancy from them were evaluated using a multivariate analysis of variance (MANOVA) according to gender (boy/girl) and age (9, 10, 11, and 12 years old). The effect size was calculated using eta squared (η^2^), and the interaction between variables using the Bonferroni statistic to determine the significance. Lastly, the Chi-square test was performed to compare body satisfaction/dissatisfaction and the desire to stay the same, gain weight, or lose weight based on age and gender. 

Analyses were performed using the statistical software package for the social sciences (SPSS, v25.0 for Windows, Armonk, New York, NY, USA). The level of statistical significance was established at *p* < 0.05 with 95% confidence.

## 3. Results

The study included 355 students, 188 (53.0%) girls, with an age range between 9 and 12 years (M = 10.53; SD = 0.84), and who were enrolled in 5th and 6th grade of Primary Education. The mean height of the participants was 1.45 m; SD = 0.09, the mean weight was 41.06 kg; SD = 10.52, and the mean BMI was 19.33; SD = 3.69. The characteristics of the participants are shown in Table 1.

Of the participants, 48% stated that they were satisfied with their body and 57.2% were dissatisfied. Of the latter, the vast majority would like to lose weight and have a figure lower than what they perceive (40.8%), and a smaller percentage of the participants (16.6%) wish they had a figure higher than what they perceived. 

Table 2 shows the results of the multivariate analysis (MANOVA), regarding perceived current figure and desired figure and differences between perceived and desired figures by students based on gender and age. 

Regarding the current perception of the body, the results indicate that there is a significant main effect in gender factor (F (1, 347) = 8.858; *p* = 0.003; η^2^ = 0.025), with boys perceiving a larger figure. A significant main effect was also found in the age factor (F (3, 347) = 3.097; *p* = 0.027; η^2^ = 0.026), the highest scores being in schoolchildren aged 10 and 12. No statistically significant differences were found in the interaction of both factors (*p* = 0.282).

Regarding the desired body, the results show that there is a significant main effect in gender factor (F (1, 347) = 20.330; *p* < 0.001; η^2^ = 0.055), with a slimmer figure being the one desired by girls more than by boys, although both want a slimmer figure than they currently perceive. A significant main effect was also found in the age factor (F (3, 347) = 2.765; *p* = 0.042; η^2^ = 0.023), with a slimmer figure being more desirable in 9-year-olds compared to 10-year-olds. No statistically significant difference was found in the interaction of both factors (*p* = 0.189).

Lastly, in the differences between the current perception of the body and the desired body, statistically significant differences were only found in the age factor (F (3, 347) = 3.381; *p* = 0.018; η^2^ = 0.028), in such a way that 9-year-old children present a higher difference than 10-year-old (*p* = 0.041) and 11-year-old (*p* = 0.018) children do. 

### 3.1. Satisfaction-Dissatisfaction with the Body 

The results obtained indicate that there are no differences with respect to gender, neither at the global level (*p* = 0.914), nor when comparing boys and girls by age (i.e., 9 years (*p* = 0.801); 10 years (*p* = 0.301); 11 years (*p* = 0.196); 12 years (*p* = 0.380)). Something similar occurs when age is compared globally, without finding statistically significant differences (*p* = 0.249). If satisfaction/dissatisfaction is compared by age only in boys, there are also no differences (*p* = 0.124); the same occurs if this comparison is made only with girls (*p* = 0.590) (Figure 1). 

### 3.2. Desire to Be Larger/Smaller

As can be seen in Figure 2, there are no statistically significant differences regarding being larger, smaller, or staying the same when compared globally based on gender (*p* = 0.797), as is the case when comparing boys vs. girls by ages (i.e., 9 years (*p* = 0.508); 10 years (*p* = 0.431); 11 years (*p* = 0.422); 12 years (*p* = 0.90)). Regarding the age of the participants, there are no differences with respect to age globally (*p* = 0.312), or when only boys are compared (*p* = 0.209) or girls (*p* = 0.365). 

## 4. Discussion

The objective of this study was to evaluate the current perception of the body versus the desired body of students and the difference between the two, depending on gender and age. Thus, the results obtained indicate that boys perceive themselves as larger than girls do, although they want to look smaller than them. More than half of the sample, 57.2%, are discontent with their bodies, with the vast majority wanting to be smaller (40.8%), compared to 42.8% who seem to be satisfied and want to maintain their current figure. The preference for being larger, smaller, or staying the same according to gender and age was also analyzed, and again, no differences were found. Therefore, body dissatisfaction could approach an equal prevalence between boys and girls at the ages studied [29]. 

In general, our results indicate that boys perceive themselves as larger than girls do, revealing a score in the perception of the body higher than that of girls. These results contrast with the results of Ramos et al. [56], which indicated that boys tend to perceive themselves smaller than girls do, as girls tend to perceive themselves as larger. Thus, we must answer affirmatively to the research question in which it was questioned whether there were differences between the current perception of the body and desired body based on gender.

Girls yearn for a slimmer figure [57], which might appear to indicate that they have the greatest dissatisfaction with their body [58]. However, when analyzing the degree of body satisfaction/dissatisfaction according to gender in our study, there were no differences between them, results that contrast with those found in research such as that of Latiff et al. [2]. Therefore, we must answer affirmatively to the second research question since there is body dissatisfaction in boys and girls. Bearing in mind the latter, it is convenient to reflect on the body dissatisfaction of schoolchildren, since based on the results of this research, it is no longer a question of gender [42,43]. The literature has always revealed that women show greater dissatisfaction with their bodies than their counterparts of the opposite gender [3,7,59]. These results could be due to the fact that the male gender has been studied less than the female [36,46,60]. These results make us think that body dissatisfaction, in the studied age range (9–12 years), does not depend on the gender of the schoolchildren, which may indicate that dissatisfaction and concern for body image are visible in both men and women [28,40,59] and that the rates of body dissatisfaction in both boys and girls are similar. Therefore, the data of the present study do not agree with the findings of Wang et al. [57], who found that the discrepancy with body appearance was greater in women than in men, although in slightly older schoolchildren. Thus, when asked if this body dissatisfaction is greater in boys or girls, the answer is that this body dissatisfaction is the same in boys as in girls.

On the other hand, although our data show that girls want to be smaller, both boys and girls indicate that they want to be smaller [57] and no differences were found between them. In fact, we can see that both sexes want to be smaller, although the dissatisfied perceive themselves with a larger figure than the satisfied ones do and wish to have a smaller figure [57,59]. Thus, these results discard the idea that girls have a greater preference for losing weight than boys do [2,3,4] and boys have a greater preference for seeing each other larger or more muscular [3,5,6,7]. In this way, we can see what is proposed by López et al. [58]: the psychological problem of body dissatisfaction has more repercussions than the physiological problem itself.

Regarding age, when analyzing the current perception of students, it is the 10- and 12-year-old schoolchildren who perceive themselves with a larger figure [59] compared to their 9- and 11-year-old peers. For this reason, and as in other investigations [6,22,28,29], body dissatisfaction begins in ages prior to puberty. As for the desired body, the youngest (9 years old) are those who want a smaller figure compared to the older ones. This is verified by visualizing the difference between the current perception of the body, which is generally perceived larger as age increases, and the desired body, whose values are lower than those of the students’ current perception. The disparities between the data are especially notable in students aged 9 and 12, with no differences being found in schoolchildren aged 10 and 11, which is consistent with the idea that there is a current trend towards body dissatisfaction at younger ages [6,22,28,29,61]. 

Despite this perception of a larger figure and the desire for a smaller figure, for both older and younger children, when satisfaction/dissatisfaction is analyzed, there are no differences between schoolchildren in the age range studied. Thus, our results do not coincide with those reported by Mak et al. [62], who stated that at the end of adolescence, the older they are, the greater their bodily disgust. Similarly, the data from our research confirm what was stated by Ivanović et al. [63] and Martin-Storey and Crosnoe [64]: as boys and girls enter adolescence, their self-awareness increases, their physical appearance becomes the reason for their concerns, and their body dissatisfaction increases [65,66]. Therefore, we have to answer affirmatively to the question of whether there are differences in the current perception of the body and desired body depending on age.

Regarding being larger, smaller, or staying the same, although the majority of dissatisfied schoolchildren want to be smaller, no significant difference has been found in the results in terms of age and wanting to be smaller, larger, or stay the same, regardless of age of the students. These desires can promote inappropriate behaviors, including nutritionally incorrect behaviors or compulsive physical activity [67,68].

As implications for teachers, this research could encourage Physical Education teachers to address body content that helps to counteract the impact of the strongly implemented beauty norms in our society. Although the amount of physical activity necessary to improve body appreciation is unknown, various Physical Education programs have been shown to support this progress. In other words, the data disclosed will facilitate teachers’ orientation of their teaching work and will allow Physical Education to be considered a subject that goes beyond recreational activities, since it can comprehensively educate students. Finally, and as proposals for improvement, interventions should be developed at school on body image in such a way that body dissatisfaction is reduced, media literacy is increased, and eating disorders are prevented [37,38,69,70].

Regarding the strengths of this study, we must highlight its relevance due to its multifactorial nature since it addressed the perception and desire of the body figure in a little-studied age range (preadolescence) based on the age and gender of the participants. 

Finally, it is necessary to mention the limitations of the study, such as that the scale was not validated for use with Spanish schoolchildren and that due to the limited sample size restricted to an urban area, the results of the study may have limited generalizability. Likewise, it should be noted that body image is influenced by context, culture, and ethnicity, so these results must be contextualized in the environment where the study was carried out (Northwest Spain). On the other hand, we must indicate that psychological methods have not been used to measure the perception of the body, so caution must be taken with the results of this research.

## 5. Conclusions

Our findings indicate that boys perceive themselves as having a larger figure than girls do and that this perception is far from reality. As for the desired figure, both boys and girls want to be smaller, but girls want a smaller figure. Regarding the age of students, the current perception of the body increases as the age of students increases. The 10- and 12-year-old students are the ones who perceive themselves with a larger current figure. On the contrary, 9-year-old schoolchildren are the ones who want a smaller figure. Likewise, more than half of the sample presents body dissatisfaction, the vast majority being those who want to be smaller. Despite this, there are no differences in body dissatisfaction between those who want to be larger or smaller based on gender (boys vs. girls), so the idea that it is the female gender that presents higher degrees of dissatisfaction and increased desire to be smaller is discarded. There are also no differences in dissatisfaction according to the age of the schoolchildren. Finally, it should be noted that the desire to be larger, smaller, or stay the same is similar between 9- and 12-year-old schoolchildren.

## Figures and Tables

**Table 1:** Sample characterization.

Variables		Frequency (%)
Gender	Boy	167 (47.0%)
	Girl	188 (53.0%)
Age	9 years old	46 (13.0%)
	10 years old	108 (30.4%)
	11 years old	166 (46.8%)
	12 years old	35 (9.9%)
BMI	Underweight person	6 (1.8%)
	Normal-weight person	179 (50.4%)
	Overweight person	112 (31.5%)
	Obese person	58 (16.3%)
Body Satisfaction	Satisfied	152 (42.8%)
	Slightly dissatisfied	121 (34.1%)
	Moderately dissatisfied	58 (16.3%)
	Severely dissatisfied	24 (6.8%)
Desire	Stay the same	151 (42.5%)
	Be smaller	145 (40.8%)
	Be larger	59 (16.6%)

Note: BMI: body mass index.

**Table 2:** Current figure and desired figure and differences between them based on gender and age.

Variables		Current Perception		Desired Figure		Differences	
Years Old	Gender	Mean	SD	Mean	SD	Mean	SD
9	boys	4.71	1.58	4.14	1.38	1.33	1.27
	girls	4.48	1.82	4.08	1.52	1.28	1.30
	Total	4.58	1.70	4.10	1.44	1.30	1.28
10	boys	5.66	1.29	5.18 ^$^	1.05	0.88	0.89
	girls	4.63 ^#^	1.36	4.24 ^#^	1.29	0.75	0.96
	Total	5.19	1.41	4.75 ^†^	1.25	0.82 ^†^	0.92
11	boys	5.14	1.18	4.90	1.05	0.67	0.82
	girls	4.43 ^#^	1.49	4.03 ^#^	1.28	0.92	1.03
	Total	4.73	1.41	4.39	1.26	0.81 ^†^	0.96
12	boys	5.41	1.27	5.05	0.82	0.94	0.89
	girls	5.22	1.47	4.11 ^#^	1.32	1.11	1.32
	Total	5.31 ^†^	1.36	4.48	1.29	1.02	1.12

Note. SD = standard deviation; ^†^ *p* < 0.05 differences to 9 years old; ^#^ *p* < 0.005 differences to boys; ^$^ *p* < 0.005 different between 9- and 10-years old boys.

## Data Availability

The data presented in this study are not available in accordance with Regulation (EU) of the European Parliament and of the Council 2016/679 of 27 April 2016 regarding the protection of natural persons with regard to the processing of personal data and the free circulation of these data (RGPD).
